# Three Cases of Elongated Mandibular Coronoid Process with Different Presentations

**DOI:** 10.5812/iranjradiol.4031

**Published:** 2014-01-30

**Authors:** Mehmet Ilguy, Pinar Kursoglu, Dilhan Ilguy

**Affiliations:** 1 Department of Dentomaxillofacial Radiology, Yeditepe University Faculty of Dentistry, Istanbul, Turkey; 2 Department of Prosthodontics, Yeditepe University Faculty of Dentistry, Istanbul, Turkey

**Keywords:** Mandible, Cone-Beam Computed Tomography, Mouth

## Abstract

Abnormal elongation of the mandibular coronoid process is rare and its etiology is not yet elucidated. The aim of this report is to demonstrate and discuss the relationship between elongated mandibular coronoid process and limitation of mouth opening with cone beam computed tomography. Although the clinical characteristic of elongation of the coronoid process is mandibular limitation, in this report, one case had problem with mouth opening. Axial scans revealed that the distance between the coronoid process and the inner face of the frontal part of the zygomatic bone may cause limitation in mouth opening. In conclusion, instead of the length, the distance between the coronoid process and the inner face of the frontal part of the zygomatic bone may be the actual reason for limitation of mouth opening. This may prevent misdiagnosis.

## 1. Introduction

Abnormal elongation of the mandibular coronoid process, formed of histologically normal bone without any synovial tissue around it, is suggestive of hyperplasia (1). Elongation of the mandibular coronoid process strikes against the zygomatic arch during mandibular movement that leads to painless difficulty in opening the mouth (2). The etiology of elongation is not yet elucidated, but several theories have been postulated, including hyperactivity of the temporal muscle that causes reactive elongation of the coronoid process and dysfunction of the temporo-mandibular joint (TMJ) caused by chronic disc displacement that would be related with cases of unilateral hyperplasia and is mentioned as one of the causes of Jacob’s disease. Other causes may include endocrine stimuli, traumatism and even genetic and family factors, osteochondroma, exostosis, osteoma and developmental alterations (1). Clinical characteristics of the mandibular coronoid process elongation are painless mandibular limitation in all movements, especially those during mandibular protrusion. Diagnosis is usually made based on the patient’s history and radiographic findings (2, 3). Cone beam computed tomography (CBCT) is a technique that produces 3-D digital imaging at reduced cost and less radiation for the patient than traditional computed tomography scan (4). Studies have suggested that CBCT provides accurate and reliable linear measurements for reconstruction and imaging of dental and maxillofacial structures (5, 6).

The aim of this report is to discuss and demonstrate the relationship between elongated coronoid process and limitation of mouth opening using CBCT.

## 2. Case Presentation

### 2.1. Case 1

**Figure 1.**
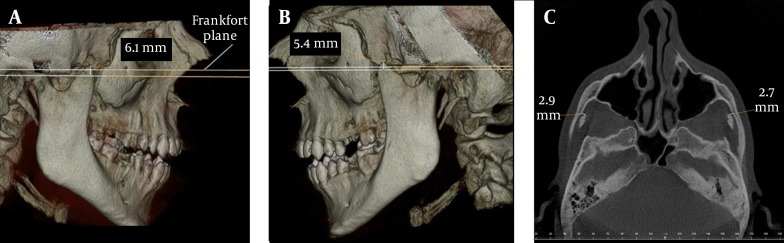
Case 1; A, 3D view of the elongated right coronoid process; B, 3D view of the elongated left coronoid process; C, The distance between the coronoid process and the inner face of the frontal part of the zygomatic bone on axial CBCT scan

A 26-year-old male patient was referred to our dental clinic with limitation of mouth opening ever since childhood. His medical anamnesis was unremarkable. He had no musculoskeletal anomalies, congenital bone dysplasia or acromegaly. Clinical examination revealed a 15 mm measurement of mouth opening. In panoramic radiography, elongation of the coronoids could not be seen clearly. For a further diagnosis, digital images with CBCT were taken using an ILUMA CBCT scanner (Imtec Corporation, OK, USA) with an amorphous silicon flat-panel image detector and a cylindrical volume of reconstruction up to 19×24 cm^2^. Images were obtained at 120 kVp, 3.8 mA, and a voxel size of 0.2 mm, with an exposure time of 40 seconds. Axial scans and 3D reconstructions were created by reformatting the axial CBCT scans on a local workstation using the ILUMA dental imaging software in accordance with the manufacturer's instructions. Before imaging, written informed consent forms were obtained from the patients. Elongation of the mandibular coronoid processes were easily detected bilaterally in both 3D images (Figure 1A and B) and axial scan (Figure 1C). For the measurements, the orientation of the 3D view was made based on the Frankfort plane (a line passing horizontally from the superior border of the external auditory meatus to the inferior border of the orbital rim). A second line passing from the top of the condyle that was parallel to the Frankfort plane was used for the length of the coronoid. Vertical measurement between the second line and the top of the coronoid process revealed the length of the elongated coronoid (Figure 1A and B). The length of the coronoid was measured as 6.1 mm on the right and 5.4 mm on the left side. On the axial scan, the distance between the coronoid process and inner face of the frontal part of the zygomatic bone was 2.9 mm on the right and 2.7 mm on the left side. The patient was informed about the treatment plan and referred to a surgeon for operation. 

### 2.2. Case 2

**Figure 2.**
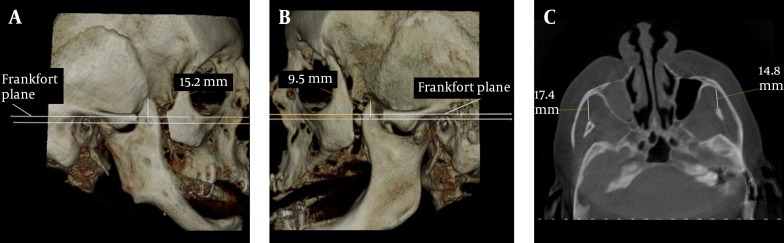
Case 2; A, 3D view of the elongated right coronoid process; B, 3D view of the elongated left coronoid process; C, The distance between the coronoid process and the inner face of the frontal part of the zygomatic bone on axial CBCT scan

A 72-year-old female patient was referred to our dental clinic for implant planning. Digital images with CBCT were taken using the same machine as the first case. During the routine evaluation of the alveolar bone, elongation of the coronoid process on both sides was observed by coincidence. Her medical anamnesis only revealed hypertension. She experienced no difficulty in opening her mouth. According to 3D view (Figure 2A and B) elongation of the mandibular coronoid processes were observed bilaterally. The lengths of the coronoids were measured as 15.2 mm on the right and 9.5 mm on the left side. On the axial scan, the distance between the coronoid process and the inner face of the frontal part of the zygomatic bone was 17.4 mm on the right and 14.8 mm on the left side (Figure 2 C). 

### 2.3. Case 3

**Figure 3.**
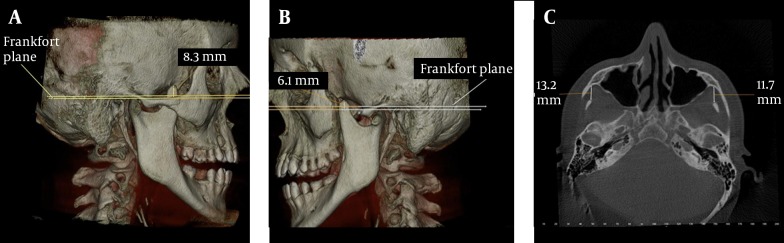
Case 3; A, 3D view of the elongated right coronoid process; B, 3D view of the elongated left coronoid process; C, The distance between the coronoid process and the inner face of the frontal part of the zygomatic bone on axial CBCT scan

A 28 year-old female patient with the chief complaint of a draining sinus tract on the right side of the mandible was referred to our clinic. The general systemic anamnesis did not reveal any major illness. She reported that she had only undergone an operation for scoliosis when she was 17 years old. There was no limitation of mouth opening. Further examination using a CBCT was performed for cysts and elongation of the coronoid processes were detected bilaterally by coincidence. The lengths of the coronoids were 8.3 mm on the right and 6.1 mm on the left side (Figure 3 A and B). On the axial scan, the distance between the coronoid process and the inner face of the frontal part of the zygomatic bone was 11.7 mm on the right and 13.2 mm on the left side (Figure 3 C). 

## 3. Discussion

In dentistry, new radiological techniques for diagnostic assessment and guidelines to select the appropriate radiographic procedures for patients suspected of having dental and maxillofacial diseases are available (7). Previous studies of TMJ assessment (8-10), and pre- and postoperative assessment of craniofacial and dentoalveolar fractures with CBCT have been reported (11, 12). Three-dimensional imaging allows us to visualize the third dimension while at the same time, it eliminates superimpositions. Elongation of the coronoid processes were previously evaluated using magnetic resonance and CT imaging (13, 14). CBCT has less radiation exposure than conventional CT and the effective dose of radiation is significantly reduced by up to 98% compared with “conventional” fan-beam CT systems (15). Bilateral hyperplasia of the coronoid processes of the mandible is quite infrequent and affects mostly males between the ages of 14 and 16 with a male to female ratio of 5:1 (16). In this report, only case 1 overlaps this ratio, whereas cases 2 and 3 were females.

The measurements of normal maximum opening, interincisal distance of the maxillary and mandibular teeth, are considered to vary between 40 and 60 mm (17), and the measurements of laterality and protrusion should be around 9 mm (18). When measurements are well beyond mean values, this may be related to muscular and intra-articular problems as well as ankylosis, neoplasia, inflammations, structural alterations and other factors. When there is a limited mouth opening, coronoid process locking could be overlooked as a cause, because interest is generally focused on the joint. Multiple factors are involved in mandibular hypomobility. An elongated coronoid process is one of these factors (3). During mouth opening, the coronoid process moves to an anterior and inferior position with the mandible. In this report, only case 1 had limited mouth opening with the shortest measurement on the axial scan (Figure 2A). In cases 2 and 3, the coronoid processes were not close to the zygomatic bone (Figure 2C and 3C). This may be the explanation of why these patients had no limitation of mouth opening. 

Coronoid process is large and projects above the level of the condyle at birth and gradually with the growth of the neck of the mandible, condyles are at a higher level in adulthood (1). Chauhan et al. reported the length of the coronoid process of a dry mandible as 2.4 cm on the right and 2.6 cm on the left side (19). In this report, the length of the coronoid process could be measured easily on 3D images. Axial scans revealed that the distance between the coronoid process and the inner face of the frontal part of the zygomatic bone may be important for limitation of mouth opening. Diagnosis of bilateral coronoid hyperplasia is often difficult to make. Elongation of the coronoid process is unfamiliar to many clinicians and may be under-reported. Initial attention is usually directed towards finding alterations of masticatory muscle function and internal derangement or ankylosis of the TMJ as the source of decreased mandibular mobility. If mouth opening is not a problem, the diagnosis could be missed without radiographic investigation. When panoramic radiography is not satisfactory for diagnosis, it could be confirmed with CBCT scans as in the reported cases. It is appropriate to use in clinical dental practice where cost and dose considerations are important and it should take part as an appropriate radiography technique in specific cases in dentistry for a well-established diagnosis. These findings should be evaluated in further cross sectional studies.

At the end a question comes into mind about whether elongation of the coronoid process really gives rise to limitation of mouth opening or is this occasion rare? Instead of the length, the distance between the coronoid process and the inner face of the frontal part of the zygomatic bone may be the actual reason for limitation of mouth opening. This may prevent misdiagnosis.
